# Factors determining the outcome of children hospitalized with severe pneumonia

**DOI:** 10.1186/1471-2431-9-15

**Published:** 2009-02-23

**Authors:** Karalanglin Tiewsoh, Rakesh Lodha, Ravindra M Pandey, Shobha Broor, M Kalaivani, Sushil K Kabra

**Affiliations:** 1 Department of Pediatrics, All India Institute of Medical Sciences, New Delhi 110029, India; 2 Department of Biostatistics, All India Institute of Medical Sciences, New Delhi 110029, India; 3 Department of Microbiology, All India Institute of Medical Sciences, New Delhi 110029, India

## Abstract

**Background:**

Pneumonia is one of the leading causes of morbidity and mortality in under fives. We carried out a comprehensive study to identify factors influencing both mortality and morbidity for children less than 5 years of age hospitalized with severe pneumonia.

**Methods:**

200 hospitalized children aged 2–60 months with World Health Organization (WHO) defined severe pneumonia were enrolled in the study. The children were managed using a standard protocol. They were closely followed up for need for change in antibiotics, prolonged hospital stay, need for mechanical ventilation and mortality. Data on the factors influencing the outcome were collected.

**Results:**

Of 200 children enrolled in the study, 113 (56.5%) needed a change in antibiotics, 102 (51%) stayed for more than 5 days in the hospital, 41 (20.5%) needed mechanical ventilation and 21 (10.5%) died. On multivariate analysis, lack of exclusive breastfeeding [RR (95%CI) 2.63 (2.16–2.86)], overcrowding [RR (95%CI) 1.94 (1.35–2.38)] and an abnormal chest x-ray [RR (95%CI) 2.29 (1.22–3.44)] were associated with the need for change of antibiotics. Lack of exclusive breastfeeding [RR (95%CI) 2.56 (2.0–2.93)], overcrowding [RR (95%CI) 2.59 (1.78–3.23)] and an abnormal chest x-ray [RR (95%CI) 2.99 (1.65–4.38)] were identified as determinants for prolonged hospital stay. Head nodding [RR (95%CI) 8.34 (2.71–12.77)], altered sensorium [RR (95%CI) 5.44 (1.34–17.56)], abnormal leukocyte counts [RR (95%CI) 5.85(1.36–17.14)] and pallor [RR (95%C) 10.88 (2.95–20.40)] were associated with mortality. Head nodding (RR (95% CI) 4.73 (1.50–6.36)] and cyanosis (RR (95%CI) 5.06 (1.80–11.34)] were the determining factors for mechanical ventilation.

In radiographically confirmed pneumonia, the determining factors for change of antibiotics were: lack of exclusive breast feeding [RR (95% CI) 2.05 (1.69–2.2)] and low birth weight [RR (95% CI) 1.59 (1.1–1.89)]. For prolonged hospital stay, the factors identified were mothers' education less than graduation [RR (95% CI) 1.5 (1.19–1.7)], lack of exclusive breast feeding [RR (95% CI) 1.77 (1.19–2.09)] and oxygen saturation of < 90% at time of presentation [RR (95% CI) 2.06 (1.42–2.42)]. Determinants for mechanical ventilation were mothers' education less than graduation [RR (95% CI) 3.6 (1.15–6.3)] and cyanosis at presentation [RR (95% CI) 10.9 (1.56–18.9)]. For mortality, the only determinant was pallor [RR (95% CI) 10.54 (1.8–21.79)].

**Conclusion:**

Children hospitalized with severe community acquired pneumonia [as defined by World Health Organization (WHO)] who had not received exclusive breast feeding, had stayed in an overcrowded homes and had an abnormal chest radiograph were more likely to fail to respond with primary antibiotic regimen and require change of antibiotics and prolonged hospital stay.

In children with radiographically confirmed pneumonia, lack of breast feeding and low birth weight was associated with need for change in antibiotics.

## Background

Pneumonia is one of the leading causes of morbidity and mortality in children under five years of age. Recent estimates from the World Health Organization suggest that pneumonia is responsible for 20% of deaths in the above age group, leading to 3 million deaths per year. Of these deaths, two thirds occur during infancy and more than 90% occur in the developing countries [1,2]. In India, recent estimates in under-fives suggest that 13% of deaths and 24% of National Burden of Disease is due to pneumonia [3]. Hospital based studies have reported that 20–30% of admissions in under-fives are due to pneumonia. Case fatality rates in hospitalized children are reported to be between 8.7–47% [4-7].

Various interventions have been done to reduce pneumonia related morbidity and mortality. In 1983, the World Health Organization (WHO) initiated the Acute Respiratory Infection (ARI) control program which led to a decline in the infant mortality rate by 10.7 (4.8–16.7) deaths per 1000 live births and decline in the mortality of under-fives by 36 deaths per 1000 live births [8]. The ARI control program includes identification of children with pneumonia by clinical features (rapid respiration and difficulty in breathing) and administration antimicrobials with a presumption that majority of pneumonia in developing countries are because of bacterial pathogens. For further impact on morbidity and mortality, a thorough knowledge of the determining factors affecting the outcome of the disease is important. Various studies have evaluated the determinants for mortality in children due to pneumonia [9-17]. Factors associated with increased mortality include young age of the mothers and lack of proper education of the father, young age of the child, late hospitalization with cyanosis, altered sensorium, grunting, associated chest indrawing, hepatomegaly, acute malnutrition, inability to drink, associated loose stools or heart disease, anemia, rickets and lack of breastfeeding [9-17].

There is a need to evaluate other relevant outcomes including the need for change of antibiotics, prolonged hospital stay and need for mechanical ventilation. We, therefore, conducted a study to evaluate factors determining both morbidity and mortality in children less than 5 years of age admitted with severe pneumonia.

## Methods

The study was conducted in the Pediatrics Wards at All India Institute of Medical Sciences (AIIMS), New Delhi, which is an urban, tertiary care center (also admits children at first presentation if they require hospitalization), between May 2004 and February 2006. Children of either sex, between 2–60 months of age hospitalized with severe community acquired pneumonia (CAP) fulfilling the WHO criteria of severe and very severe pneumonia [18] were enrolled within 24 hours of admission. The case definition of severe and very severe pneumonia included children presenting with cough with difficulty in respiration, having increased respiratory rate (children < 2 months; > 60/minute, 2–11 months, > 50 per minute and 13 to 60 months, > 40 per minute) with chest indrawing with or without inability to drink or cyanosis. Children with co-morbidities like meningitis or chronic respiratory diseases, those who were admitted in another hospital prior to presentation or whose parents did not give consent were excluded.

Parents of patients who met the inclusion criteria were invited to participate in the study. A pre-designed performa was used for recording the details of personal and demographic profile, clinical presentation, underlying disease, laboratory investigations, treatment and course in the hospital and the final outcome. Relevant past and family history was enquired and recorded. The number of people staying together and the number of available rooms in the house was recorded. Overcrowding was determined by calculating number of family members per room [19]. A child who was not found to be in the following norm was labeled as staying in overcrowded house.

1 Room 2 persons

2 Rooms 3 persons

3 Rooms 5 persons

4 Rooms 7 persons

5 or more rooms 10 persons (additional 2 for each further room)

Immunization status was assessed by verifying the immunization records or asking the parents. The mode of delivery, any prenatal insult and birth weight of the child was recorded.

Dietary history was obtained from parents. Details of breast feeding and addition of top feeds was recorded. An infant who received only breast feeding till age of 6 months was considered to be exclusively breast fed. Lack of exclusive breast feeding was defined as receipt of any top feed in the first 6 months of life. For children above 6 months of age and those not on breast feeding, the caloric intake was recorded by 24 hour recall.

A detailed clinical examination was done at the first contact. Results of the investigations including complete blood count, chest x-ray, blood culture, serum electrolytes and arterial blood gas analysis were recorded.

Chest radiographs were performed in all the patients. The chest radiograph were read by one of the authors (KT, RL, SKK) and interpreted as normal or showing consolidation, infiltrates or pleural effusion. In addition, a nasopharyngeal aspirate was taken for identification of viruses: respiratory syncitial virus, adenovirus, para influenza viruses 1, 2, 3, and influenza viruses A and B by Direct Fluorescence assay (Respiratory panel 1, viral screening and identification, DFA kit, Chemicon) [20].

Our primary objective was to determine the association between the various clinical and laboratory parameters and the need for change in antibiotics in children with WHO defined severe pneumonia. Secondary objectives were to find out the determining factors that were associated with prolonged stay in the hospital (defined as hospitalization exceeding 5 full days), factors associated with mortality and need for mechanical ventilation.

A protocol for antibiotic therapy (Additional file 1, Table S1) was circulated to all the clinicians for the uniform treatment of children with pneumonia [21]. The treatment given and progress notes were recorded daily including any change of antibiotics by investigators (KT, SKK). Day of discharge or death was recorded.

The antibiotics were changed if the patient did not improve after 48 hours of initiation of treatment or deteriorated in form of increasing chest indrawing or worsening hypoxemia (worsening oxygen saturation). Mechanical ventilation was indicated if the child had respiratory failure (PO_2 _<60 mm Hg on supplemental oxygen or PCO_2 _> 50 mm Hg) or had impending respiratory failure (marked respiratory distress, child likely to tire out). Discharge was considered when the child's respiratory rate reduced below the age-specific cut-off, with absence of chest indrawing, hypoxemia and fever for at least 24 hours.

### Statistical analysis

The method of sample size calculation for prospective observational study was used. To estimate a rate of change of antibiotics of 40% with 95% confidence, a sample size of 188 was calculated to estimate the same with a precision of 7%. For estimating the relative risk for a factor (determinant) as 2, a sample size of 108 was calculated for 80% power and 95% confidence, assuming the rate of change of antibiotics in the group without the determining factor as 30%, and the ratio of exposed to unexposed as 1:2 [22].

The data collected were entered in Epi Info 2000 and then transferred to STATA 9.0 (StataCorp LP, College Station, Texas, USA) for statistical analysis. Chi Square test was used to study the associations between the determining factors and the outcome. The significant factors derived from the bivariate analysis were studied separately using unadjusted relative risk (95% confidence interval). To find out the independent contribution of each factor towards the outcome, we used the multiple logistic regression analysis. From the calculated adjusted odds ratio (95% confidence interval), we derived adjusted relative risk using a method published by Jhang and Yu [23]. The p value of less than 0.05 was considered statistically significant.

We analyzed our outcome variables in children with radiographically confirmed pneumonia after excluding children with normal chest radiographs and with congenital heart diseases.

The study was approved by the faculty of the Departmental of Pediatrics, AIIMS, New Delhi. A written informed consent was taken from parents.

## Results

### Factors determining the outcomes in children with community acquired pneumonia diagnosed on the basis of WHO case definition

A total of 200 children met the inclusion criteria and did not have exclusion criteria. All the 200 {mean age (SD) 11.74 months (12.44)} children were enrolled in the study. 143 (71.5%) were infants less than 12 months of age, 127 (63.5%) were boys, 136 (68%) came from overcrowded families, 119 (59.5%) had received exclusive breast-feeding. 170 (85%) patients had a sterile blood culture; 12 samples grew *Streptococcus pneumoniae*, while others included *Staphylococcus aureus *in 8, Acinetobacter in 6, Enterobacter in 2, Klebsiella in 1 and Pseudomonas species in 1. Out of the 139 patients whose nasopharyngeal aspirate was taken, 11 were positive for viruses- 5 for influenza A, 1 for influenza B, 1 for adenovirus, 2 each for para-influenza 1 and 3. Serum sodium values were within normal limit (142 ± 5.8 mEq/l) in all except one patient who had serum sodium of 129 mEq/L. In 146 (83%) children, chest radiograph showed consolidation or infiltration. The other baseline characters and frequency of clinical and laboratory parameters of the participants are shown in Additional file 1, Table S2.

Of the 200 patients, 113 (56.5%) needed a change of antibiotics because of worsening of hypoxia and/or chest indrawing. No antibiotic changes were needed for 'no improvement' in clinical features at 48 hours. A total of 102 (51%) stayed in the hospital for more than 5 days, 41(20.5%) needed mechanical ventilation and 21 (10.5%) died. Ten patients died within 24 hours of hospitalization, 4 died between 24–48 hours and 7 died after 48 hours.

On multivariate analysis, factors associated with treatment failure requiring change of antibiotics included overcrowding at home [RR (95% CI)- 1.94 (1.35–2.38)], lack of exclusive breastfeeding [RR (95% CI)- 2.63 (2.16–2.86)] and an abnormal chest radiograph [RR (95% CI)- 2.29 (1.22–3.44)] (Additional file 1, Table S3).

Factors associated with prolonged hospital stay included overcrowding at home [RR (95%CI)- 2.59 (1.78–3.23)], lack of exclusive breastfeeding [RR (95%CI)- 2.56 (2.0–2.93)] and an abnormal chest radiograph [RR (95%CI)- 2.99 (1.65–4.38)] (Additional file 1, Table S4) on multivariate analysis.

For mortality in children with severe pneumonia, head nodding (head nods with each respiration due to contractions of accessory muscles of respiration including sternomastoid) [RR (95%CI)- 8.34 (2.71–12.77)], altered sensorium (incoherent and not interested in surrounding) [RR (95%CI)- 5.44 (1.34–17.56)], abnormal leukocyte counts [RR (95%CI)- 5.85 (1.36–17.14)] and pallor [RR (95%CI)- 10.88, (2.95–20.40)] were identified as determinants on multivariate analysis (Additional file 1, Table S5).

The determining factors for mechanical ventilation identified on multivariate analysis were cyanosis [RR (95%CI)- 5.06 (1.80–11.34)] and head nodding [RR (95% CI)- 4.73 (1.50–6.36)] at admission (Additional file 1, Table S6).

### Factors determining the outcomes in children without underlying heart disease with radiographically confirmed pneumonia

A total of 127 children {mean age (SD) 11.74 months (12.44)} had radiographically confirmed pneumonia and no evidence of congenital heart disease. 87 (69%) were infants less than 12 months of age, 85 (67%) were boys, 93 (73%) came from overcrowded families, 57 (45%) did not receive exclusive breast-feeding. 106 (83%) patients had a sterile blood culture. Out of 21 positive cultures 10 samples grew *Streptococcus pneumoniae*, while others included *Staphylococcus aureus *in 7, Acinetobacter in 1, Enterobacter in 1, Klebsiella in 1 and Pseudomonas species in 1. Nasopharyngeal swabs were collected in 105 children with abnormal radiographs; none of them were found to be positive for viruses. The other baseline characters and frequency of clinical and laboratory parameters of the participants are given in Additional file 1, Table S7.

Of the 127 patients, 84 (66%) needed a change of antibiotics due to worsening of hypoxia and/or chest indrawing. None of them needed change because of 'no improvement' in clinical features at 48 hours. A total of 80 (63%) stayed in the hospital for more than 5 days, 28 (22%) needed mechanical ventilation and 13 (10.2%) died. Nine children died within 24 hours of admission, one between 24–48 hours and 3 died after 48 hours of admission. There was no significant difference in these outcome parameters between children with WHO defined pneumonia or radiographically confirmed pneumonia.

On multivariate analysis, factors associated with treatment failure requiring change of antibiotics included lack of exclusive breastfeeding [RR (95% CI)- 2.05 (1.69–2.2)] and birth weight of < 2.5 Kg [RR (95% CI)- 1.59 (1.1–1.89)] (Additional file 1, Table S8).

Factors associated with prolonged hospital stay included mothers education less than graduation [RR (95%CI)- 1.5 (1.19–1.7)], lack of exclusive breastfeeding [RR (95%CI)- 1.77 (1.19–2.09)] and oxygen saturation of less than 90% at time of presentation [RR (95%CI)- 2.06 (1.42–2.42)] (Additional file 1, Table S9) on multivariate analysis.

For mortality in children with severe pneumonia, presence of pallor at presentation [RR (95%CI)- 10.54 (1.8–21.79)] (Additional file 1, Table S10).

The factors determining need for mechanical ventilation identified on multivariate analysis were cyanosis at presentation [RR (95%CI)- 10.9 (1.56–18.9)] and mothers education less than graduation [RR (95% CI)- 3.6 (1.15–6.3)] (Additional file 1, Table S11).

## Discussion

We conducted a cohort study to identify the clinical and laboratory parameters associated with adverse outcomes in under-five children hospitalized with WHO defined community acquired pneumonia. We observed that overcrowding, lack of exclusive breastfeeding and an abnormal chest radiograph were associated with the risk for need of change of antibiotics. These three factors were also associated with increased hospital stay. Head nodding, altered sensorium, abnormal leukocyte counts and pallor were identified as determinants associated for death. Cyanosis and head nodding at admission were associated with need of mechanical ventilation. Demographic profile, passive smoking, malnutrition etc were not found to be significant factors determining the outcome of hospitalized children with WHO defined severe pneumonia.

We planned to include all the children (including children with heart diseases, immune deficiency etc) presenting with WHO defined community acquired pneumonia who had not received antibiotics and were not in hospital to document whether these illnesses led to risk of treatment failure in these children. Though we did not have any child with primary immune deficiency, but congenital heart disease, associated loose stools or severe malnutrition did not emerge as factors predisposing them for change in antibiotics, prolonged hospital stay, need for mechanical ventilation or mortality. This finding of our study suggests that children with associated problems (heart disease, loose stools or severe malnutrition) can be managed with common protocol for community acquired pneumonia.

The primary outcome was the need for change in antibiotics in children with WHO defined severe pneumonia. Decision to change the antibiotics was based on clinical features; no attempt was made to document bacterial infection before changing the antibiotics as it is difficult. First line antibiotics received by our patients included: ampicillin + gentamicin, or amoxicillin-clavulanic acid. These are reasonably broad spectrum antibiotics to cover common pathogens responsible for pneumonia in children. All our patients required change in antibiotics because of worsening (chest indrawing or hypoxia) during the course of illness. None of the patient required antibiotic change due to lack of improvement at 48 hours; suggesting that they had possibly infection due to drug resistant organisms and responded to change of antibiotics. Reports from this part of world suggest increasing resistance to common antibiotics including ampicillin [24,25].

There are no previous reports on outcome measures in terms of need of change in antibiotics, prolonged hospital stay or need for ventilator support. We observed a mortality rate of 10.5% which compares with the reported case fatality rate of 9.8% [13] and 10.45% [4] in similar settings.

Previous studies report lack of exclusive breastfeeding increases the risk of both upper and lower respiratory tract illness. Lack of breast feeding has been reported to be associated with increase risk of development of severe pneumonia by 1.5 to 2.6 times [26-29]. In the present study, lack of exclusive breastfeeding was identified to be an important determinant associated with the need for change in antibiotics and prolonged hospital stay in severe pneumonia.

The mechanisms by which breast-feeding provides protection against respiratory infections are incompletely understood. In addition to passive protection, breast milk seems to affect the infant's systemic immune system via multiple mechanisms including maturational, anti-inflammatory, immuno-modulatory and antimicrobial action [30]. Changes in immune phenotype after exposure to maternal milk, including increase in post-vaccination interferon-α levels and in natural killer cell numbers could result in prolonged protection against respiratory infections [31,32]. In addition, there is experimental evidence in animals that maternal milk lymphocytes cross the infant's intestinal wall and enter the circulation [33]. It is postulated that these cells activate the infant's immune system. Anti-inflammatory cytokines such as interleukin-10 and transforming growth factor β are also present in maternal milk and taken up by neonatal tissues, in which they are associated with a decrease in inflammatory immune responses [34] and augmented secretary immunoglobulin A synthesis [35,36]. These findings of infant immune responses associated with breastfeeding lend biological credence to the findings in our study, in that; breastfeeding could result in a decrease in respiratory infections for many months or even years [37].

There are limited number of studies that have explored the association between breast feeding and outcome of pneumonia. Yoon et al had concluded that lack of breast feeding increased the rate of mortality by 5.7 times in children with acute lower respiratory infection and diarrhea in the first 6 months of life [14]. Victoria et al had reported that lack of breastfeeding was associated with 3.2 times more risk of death from pneumonia [17]. We did not observe lack of breast feeding as a determining factor for mortality in our study. This may be due aggressive interventions like change of antibiotics and mechanical ventilation.

While the influence of crowding on the risk of pneumonia is well understood, there are only a few studies documenting influence of crowding on the outcome of pneumonia in children. The possible mechanism may be influence of crowding on the selection and spread of resistant bacteria [38,39]. These observations may explain the association of crowding with need for change in antibiotics and prolonged hospital stay. Shah et al had shown that sharing of bedroom by more than two persons increases the risk of severe pneumonia by 1.8 times. [28].

The other independent factor associated with the morbidity of severe community acquired pneumonia in under-fives in our study was an abnormal chest radiograph. These included those showing infiltrates or consolidation. An abnormal chest radiograph was more likely to be due to bacterial infection and need either a change of antibiotics or a prolonged stay in the hospital. However, when we compared radiographically confirmed and WHO defined pneumonia we did not find significant difference in the main outcome. Djelantik et al in their study also demonstrated that radiological findings were not significant factor determining the outcome [9].

In the present study, head nodding at admission- a sign of severe respiratory distress, abnormal leukocyte counts, cyanosis, pallor and altered sensorium were identified to be determining factors associated with mortality and need for mechanical ventilation. Head nodding, high leukocyte counts and altered sensorium are all indicators of severity of illness and therefore, may be associated with mortality. Demers et al reported that altered sensorium in children with acute respiratory tract infection is associated with increased risk of mortality by 3.2 times [10]. Pallor, which could be present due to anemia or shock, was identified to be a significant determining factor affecting mortality. Banejeh et al had identified anemia as a significant risk factor increasing mortality from pneumonia by 5.8 times. [14].

For need of mechanical ventilation, head nodding and cyanosis at admission were identified as significant risk factors. There are no earlier reports that studied risk factors for need of mechanical ventilation. Therefore, presence of head nodding and cyanosis, markers of severity of the disease indicate need for admission to an intensive care unit.

After excluding children with normal radiographs and heart disease, the determining factors for change of antibiotics were: lack of exclusive breast feeding and low birth weight. For prolonged hospital stay, the determinants identified were mothers education less than graduation, lack of exclusive breast feeding and oxygen saturation of less than 90% at time of presentation. Determinants for mechanical ventilation were mothers' education less than graduation and cyanosis at presentation. For mortality, the only determining factor was pallor.

In a recent study on WHO defined community acquired non-severe pneumonia; chest radiographs were reported as normal in 82% of children. In the present study, normal chest radiographs were observed in 54 (27%) of patients with severe pneumonia. Blood cultures were more common in children with radiographically confirmed pneumonia and viruses were isolated only from those with normal chest radiographs. These findings suggest that there is need to improve WHO case definition of pneumonia as majority of children with normal chest radiographs were suffering from viral infections [40].

Two parameters including low birth weight and maternal education emerged as determinants of outcomes in radiographically confirmed pneumonia. Rest of the determining factors were common with the analysis of clinical severe pneumonia. Low birth weight has been associated with development of severe pneumonia [28,41]. Low birth weight has also been reported to be a risk factor for increased mortality [15].

Maternal education emerged as an important determinant in radiographically confirmed pneumonia for prolong hospital stay and need for mechanical ventilation. There are not reports linking maternal education with the outcome of pneumonia. However, maternal knowledge of symptoms of pneumonia is associated with early recognition and utilization of health care facilities for their children [42,43]. Only possible explanation may be that educated mothers identified illness in their children early and availed early treatment.

Proportionately more children died within 24 hours of hospitalization in children who had radiographically confirmed pneumonia, suggesting that these patients were having bacterial pneumonia and could have been saved only if intervention could have been done early. Though in our study delayed reporting to the health care facility did not emerge a significant risk factor but Banajeh et al [13] reported late hospitalization with cyanosis as one of the significant risk factor for mortality.

Strengths of the present study include prospective nature of the study and that the patients were enrolled and multiple factors were observed daily till the end point occurred. Limitations of the study were that the bacterial etiology was based only on blood cultures. Blood culture positivity was only between 5–10%. For identification of bacteria we did not perform serological tests or surrogate markers like serum C-reactive protein or procalcitonin. Our aim in the present study was to see if we could identify children with WHO defined severe pneumonia who require change of antibiotics or prolong hospital stay. WHO case definition is for countries with infant mortality of > 40/1000 live birth. These guidelines recommend administration of antibiotics to all children with pneumonia. It does not differentiate between bacterial and viral pneumonia as it is not possible to do so by clinical features alone. However, the observation that failures were associated with abnormal chest radiograph and very few nasopharyngeal aspirates were positive for viruses suggest that most of our patients were suffering from bacterial pneumonia.

## Conclusion

Children with severe community acquired pneumonia having lack of exclusive breast feeding, coming from overcrowded homes and having abnormal chest radiograph with low birth weight are less likely to respond to first line antibiotics and need a change of antibiotics; therefore, may be treated aggressively with second line antibiotics from the beginning that may reduce their hospital stay. Children with evidence of severe respiratory distress such as cyanosis, head nodding and altered sensorium and with pallor or high leukocyte counts are at greater risk of mortality and should preferably be admitted in a pediatric intensive care unit for more aggressive monitoring and management that could reduce their mortality.

## Competing interests

The authors declare that they have no competing interests.

## Authors' contributions

KT: Involved in planning of study, collected data, helped in analysis of data and drafted manuscript, RL: Involved in planning of study, analysis of data and manuscript writing, RMP: Involved in planning of study, and participated in data analysis, SB: Involved in investigations for viral etiology of pneumonia, MK: Participated in analysis of data and manuscript writing, SKK: Involved in planning of study, participated in data analysis and manuscript writing. He will act as guarantor for the paper. All authors read and approved the final manuscript.

## Pre-publication history

The pre-publication history for this paper can be accessed here:



## Supplementary Material

Additional file 1 **11 Additional files**1, **Table Ss.** Click here for file
