# Supplementary material for: Factors determining the outcome of children hospitalized with severe pneumonia
Source: BMC Pediatr. 2009 Feb 23;9:15. doi: 10.1186/1471-2431-9-15 (PMC2651138; doi:10.1186/1471-2431-9-15)
Supplement: Additional file 1 — 11 Additional files1, Table Ss. [file 1471-2431-9-15-S1.doc]

**Table 1: Antibiotic protocol**

^Antibiotics^ **Severe community acquired pneumonia**

First line Chloramphenicol;

Penicillin / Ampicillin + Gentamicin

**_________________________________________________________________________________**

Alternative Cefuroxime ;

Amoxicillin clavulinic acid

**_________________________________________________________________________________**

Second line Amoxicillin clavulinic acid + Aminoglycoside

Cefuroxime +Aminoglycoside

**_________________________________________________________________________________**

Alternative Chloramphenicol +cephazolin

**_________________________________________________________________________________**

Modified from reference 20. Antibiotics are started by intravenous route

Table 2: Risk factors and their frequency in the study subjects

Characteristics Children Children Children Children needing

needing change needing prolonged mechanical

of antibiotics^1^  hospital stay^2^ ventilation^3^

________________________________________________________________________________________________

N (%) Yes No Yes No Yes No

N(%) N(%) N(%) N(%) N(%) N(%)

_________________________________________________________________________________________________

Age < 12 months 143 (71.5) 81 62 74 69 18 125

(56.6) (43.4) (51.8) (483) (12.6) (87.4)

_________________________________________________________________________________________________

Sex: Males 127 (63) 71 56 66 61 12 115

(55.9) (44.1) (53.0) (48.0) (9.5) (90.6)

Mothers age <25 years 110 (55) 69* 41 65* 45 16 94

(62.7) (37.3) (59.1) (40.9) (14.6) (85.5)

Fathers age <30 years 161 (81) 98* 63 89* 72 18 143

(60.9) (39.1) (55.3) (44.7) (11.2) (88.8)

Mothers education 70 (35) 49* 21 43* 27 12 58 *

< graduation (70) (30) (61.4) (38.6) (17.1) (82.9)

Fathers education 162 (81) 30* 8 27* 11 9 29*

< graduation (79) (21) (71) (29) (23.7) (76.3)

Pets at home 19 (10) 7 12 5* 14 1 18

(36.8) (63.2) (26.3) (73.7) (5.3) (94.8)

Overcrowding 136 (68) 91* 45 86* 50 15 121

(66.9) (33.1) (63.2) (36.8) (11) (89)

Cooking fuel other 48 (24) 38* 10 32* 16 12 36*

than LPG (79.2) (20.8) (66.7) (33.3) (25) (75)

Smoking by father 70 (35) 66* 15 51* 19 11 59*

(78.6) (21.4) (72.9) (27.1) (15.7) (84.3)

No Exclusive 81 (40.5) 74* 7 66 * 15 12 69

breast feeding (91.4) (8.6) (81.5) (18.5) (14.8) (85.2)

Severely malnourished 25 (13) 17 (68) 8(32) 14(56) 11(44) 11(44) 14* (56)

Birth weight < 2.5 Kg 84 (42) 65* 19 56* 28 15 69*

(77.4) (22.6) (66.7) (33.3) (18) (82)

Incomplete immunization 56 (28) 42* 14 42* 14 14 42*

(75) (25) (75) (25) (25) (75)

First medical contact 65 (33) 54* 11 49* 16 13 52

>3 days of onset of illness (83.1) (16.9) (75.4) (24.6) (20) (80)*

Presence of Congenital 24 (12) 19* 5 14 10 8 17*

heart disease (79.2) (20.8) (58.3) (41.7) (33.3) (66.7)

Altered sensorium 68 (34) 47 21 39 29 18 50

(69.1) (30.9) (57.4) (42.6) (26.5) (73.5)

Abnormal development 18 (9) 12 6 11 7 2 16

(66.7) (33.3) (61.1) (38.9) (11.1) (88.9)

Associated loose stools 31 (16) 15 16 12* 19 6 25

(48.4) (51.6) (38.7) (61.3) (19.3) (80.6)

_________________________________________________________________________________________________

Respiratory rates 115 (58) 76* 39 69* 46 17 98*

> 70/ minute (66.1) (33.9) (60) (40) (14.8) (85.2)

_________________________________________________________________________________________________

Wheezing 47 (24) 11* 36 10* 37 1 46*

(23.4) (76.6) (21.3) (78.7) (2.1) (97.9)

Grunting/ groaning 91 (45) 72* 19 62* 29 20 71*

(79.1) (20.9) (68.1) (31.9) (22) (78)

Head nodding 15 (8) 10 5 7 8 8 7*

(66.7) (33.3) (46.7) (53.3) (53.3) (46.7)

Cyanosis 75 (38) 61* 14 53* 22 21 54*

(81.3) (18.7) (70.7) (2.3) (28) (72)

Oxygen saturation 124 (62) 89* 35 81* 43 21 103*

< 90% at arrival (71.8) (28.2) (65.3) (34.7) (16.9) (83.1)

Hemoglobin <8 g/dL 20 (10) 11(55) 9(45) 8 (40)* 12 (60) 10 (50) 10 (50)

Abnormal total 90 (45) 52* 58 54* 36 18 72*

leukocyte counts (47.3) (52.7) (60) (40) (20) (80)

Abnormal Chest X-ray 146 (83) 99* 47 90* 56 20 126*

(67.8) (32.2) (61.6) (38.4) (13.7) (86.3)

Blood culture positive 30 (15) 26* 4 24* 6 5 25*

(86.7) (13.3) (80) (20) (16.7) (83.3)

Virus positive in 11 (6) 5 6 5 6 0 11

Nasopharyngeal aspirates (45.4) (54.6) (45.4) (54.6) (100)

Abnormal arterial 82 (41) 68* 14 58* 24 21 61*

blood gas analysis (82.9) (17.1) (70.7) (29.3) (25.6) (74.4)

_________________________________________________________________________________________________

P < 0.05

1. 113 required change in antibiotics

2. 102 stayed longer in hospital

3. 41: required mechanical ventilation

Table 3: Factors associated with increased risk for change of antibiotics

**Risk Factors Change of antibiotics P Unadjusted Adjusted**

Yes No **RR (95%CI) RR (95% CI)**

**_________________________________________________________________________________**

**No Exclusive breast feeding**

No- N (%) 74 (91.36) 7 (8.64) 0.0001 2.79 2.63

Yes- N (%) 39 (32.77) 80 (67.23) (2.14- 3.64) (2.16- 2.86)

**Overcrowding**

Yes- N (%) 91 (66.91) 45 (33.09) 0.0001 1.95 1.94

No- N (%) 22 (34.38) 42 (65.63) (1.36-2.78) (1.35- 2.38)

**Chest radiograph**

Abnormal- N (%) 99 (67.81) 47 (32.19) 0.0001 3.32 2.29

Normal- N (%) 10 (20.41) 39 (79.59) (1.89-5.84) (1.22-3.44)

# Table 4: Factors associated with prolonged hospital stay

Risk factors Prolonged hospital stay P Unadjusted Adjusted RR

Yes No RR (95% CI) (95% CI)

_________________________________________________________________________________

**No exclusive breast feeding**

No- N (%) 66 (81.48) 53 (58.89) 0.0001 2.53 2.59

Yes- N (%) 36 (30.25) 14 (73.68) (1.62-3.94) (1.78-3.23)

_________________________________________________________________________________

**Over crowding**

Yes- N (%) 86 (63.24) 50 (36.76) 0.0001 2.53 2.59

No- N (%) 16 (25) 84 (46.41) (1.62-3.94) (1.78-3.23)

**Chest radiograph**

Abnormal- N(%) 90 (61.64) 56 (38.36) 0.0001 3.77 2.99

Normal- N (%) 8 (16.33) 41 (83.67) (1.98-7.20) (1.65-4.38)

# Table 5: Factors associated with increased risk for need of mechanical ventilation

Risk Factors Need for mechanical P Unadjusted Adjusted

Ventilation RR (95%CI) RR (95%CI)

Yes No

**Head nodding**

Yes- N (%) 13 (86.67) 2 (13.33) 0.0001 5.73 4.73

No- N (%) 28 (15.14) 157 (84.86) (1.50- 6.36) (1.50- 6.36)

**Cyanosis**

Yes- N (%) 36 (48) 39 (52) 0.0001 12 5.06

No- N (%) 5 (4) 120 (96) (4.93-29.24) (1.80-11.34)

# Table 6: Factors associated with mortality

Risk factors Mortality P Unadjusted Adjusted

Yes No RR (95%CI) RR (95%CI)

**Head nodding**

Yes- N (%) 8 (53.33) 7 (46.67) 0.0001 7.58 8.34

No- N (%) 13 (7.03) 172 (92.97) (3.74-15.38) (2.71-12.77)

Altered sensorium

Yes- N (%) 18 (26.47) 50 (73.53) 0.0001 11.64 5.44

No- N(%) 3 (2.27) 129 (97.73) (3.55-38.15) (1.34- 17.56)

Leukocyte counts

Abnormal- N (%) 18 (20) 72 (80) 0.0001 7.33 5.85

Normal- N (%) 3 (2.73) 107 (97.27) (2.23-24.10) (1.36- 17.14)

Pallor

Yes- N (%) 15 (26.23) 45 (73.77) 0.0001 7.29 10.88

No- N (%) 5 (3.6) 134 (96.4) (2.79-19.01) (2.95- 20.4)

Table 7: Factors and their frequency in children with radiographically confirmed pneumonia

Characteristics Number of Children needing Children needing Children needing

children Change of Prolonged hospital Mechanical

Antibiotics^1^  stay^2^ Ventilation^3^

N (%) Yes No Yes No Yes No

127 (100) N(%) N(%) N(%) N(%) N(%) N(%)

Mothers’ age 77(61) 57(74)* 20(26) 56(73)* 21(27) 22(27)* 55(73)

<25 years

Fathers’ age 100(79) 70(70) 30(30) 67(67) 33(33) 24(24) 76(76)

<30 years

Mothers’ education 52(41) 41(79) 11 (21) 39(75) 13(25) 18(35) 34(65)

< graduation

Fathers’ education 34(27) 28(82) 6(18) 25(74) 9(26) 13(38) 21(62)*

< graduation

Pets at home 10(8) 4(40) 6(60) 3(30) 7(70) 0 10(100)

Overcrowding 93(73) 70(75)* 23(25) 69(74)* 24(26) 23(25)* 70(75)

Cooking fuel 38(30) 32(84)* 6(16) 28(74) 10(26) 16(42) 22(58)

other than LPG

Smoking by father 53(42) 43(66) 10(34) 42(79) 11(21) 17(32) 36(68)

No Exclusive 57(45) 53(93)* 4(7) 49(86)* 8(14) 16(28) 41(72)

breast feeding

Severely 20(16) 14(70) 6(30) 11(55) 9(45) 8((60)* 12(40)

Malnourished

Birth weight <2.5 Kg 58(46) 50(86)* 8(14) 48(80)* 10(20) 12(24) 39(76)

Incomplete 44(37) 38 (86)* 6 (14) 35 (80)* 9 (20) 21 (48)* 23 (52)

Immunization

First medical 51(40) 43(84)* 8(16) 41(80)* 10(20) 12(24) 39(76)

contact >3 days

of onset of illness

Altered sensorium 46(36) 35(76) 11(24) 31(67) 15(33) 19(41) 27(53)

Abnormal development 12(9) 9(75) 3(25) 9(75) 3(25) 0 12(100)

Associated loose stools 19(16) 13(68) 6(32) 11(58) 8(42) 7(37) 12(63)

Respiratory rates 78(61) 58(74)* 20(26) 56(72)* 22(28) 23(30)* 55(70)

>70/ minute

Pallor 48(33) 38 (79)* 10 (21) 33 (69) 15 (31) 34 (71) 14 (29)

Wheezing 1310) 5(38) 8(62) 5(38) 8(62) 2(15) 11(85)

Grunting/ groaning 65(51) 52(80)* 13(20) 47(72)* 18(28) 23(65)* 42(35)

Head nodding 8(6) 5(63) 3(37) 3(38) 5(62) 7(81)* 1(19)

Cyanosis 54(42) 45(83)* 9(17) 40 (74)* 14 (26) 24(44)* 30(55)

Oxygen saturation 87(67) 68(78)* 19(22) 65(75)* 22(25) 28(68)* 59(32)

< 90% at arrival

Hemoglobin <8 g/dL 17(13) 10(59) 7(41) 8(47) 9(53) 5(25) 12(71)

Abnormal total leukocyte 69(54) 48(70) 21(30) 46(67) 23(33) 17(25) 52(75)

Counts

Blood culture positive 21(17) 18(86)* 3(14) 19(90)* 2(10) 5(24) 16(76)

Abnormal arterial 60(47) 50(83)* 10(17) 45(75)* 15(25) 28(47)* 32(53)

blood gas analysis

* P < 0.05;, 1. 84 children needed change of antibiotics; 2: 80 children had prolonged hospital stay; 3: 28 children needed mechanical ventilation

Table 8: Factors associated with increased risk for change of antibiotics in radiographically confirmed pneumonia

Risk Factors Change of P Unadjusted Adjusted RR

Antibiotics RR (95%CI) (95% CI)

**Yes No**

**No exclusive breast feeding**

Yes- N (%) 53(93)* 4(7) <0.0001 2.09 2.05

No- N (%) 31 (44) 39 (56) (1.59-2.75) (1.69- 2.2)

**Birth weight(<2 Kg)**

Yes- N (%) 50 (86) 8 (14) <0.0001 1.84 1.59

No- N (%) 29 (47) 33 (53) (1.38-2.45) (1.1- 1.89)

Table 9: Factors associated with prolonged hospital stay radiographically confirmed pneumonia

Risk factors Prolonged P Unadjusted Adjusted

Hospital stay RR (95%CI) (95%CI)

Yes No

**No exclusive breast feeding**

No- N (%) 49 (86) 8 (14) 0.0001 2.09 1.77

Yes- N (%) 31 (44) 39 (56) (1.59-2.75) (1.19- 2.09)

**Mothers education < graduation**

Yes- N (%) 39(75) 13(25) 0.01 1.37 1.5

No- N (%) 41 (55) 34 (45) (1.05-1.77) (1.19- 1.7)

**Oxygen saturation < 90%**

Abnormal N(%) 65(75)* 22(25) 0.001 1.99 2.06

Normal N(%) 15 (38) 25 (62) (1.31- 3.02) (1.42- 2.42)

# Table 10: Factors associated with increased risk for need of mechanical ventilation in radiographically confirmed pneumonia

Risk Factors Need for mechanical P Unadjusted Adjusted

Ventilation RR (95%CI) RR(95%CI)

Yes No

**Mothers education < graduation**

Yes-N (%) 18 (35) 34 (65) 0.004 2.59 3.6

No- N (%) 10 (13) 65 (87) (1.3-5.1) (1.15- 6.3)

**Cyanosis**

Yes- N (%) 24 (44) 30 (56) 0.0001 8.1 10.9

No- N (%) 4 (5) 69 (95) (2.98-22.01) (1.56- 18.9)

Table 11: Factors associated with mortality in radiographically confirmed pneumonia

Risk Factors Mortality P Unadjusted Adjusted

Yes No RR (95%CI) RR(95%CI)

**Pallor**

Yes- N (%) 10 (21) 38 (79) 0.0021 5.40 10.54

No- N (%) 3 (4) 76 (96) (1.58-18.94) (1.8- 21.79)
